# Utility of Vascular Endothelial Specific Peptides for Enhancement of Adeno-Associated Virus-Mediated Gene Transfer

**Published:** 2008-09

**Authors:** Juan A. Merchan, Jarrod Dean, Federico Azpurua, Sabyasachi Sen, Yan Zhu, Ryuichi Aikawa

**Affiliations:** *Cardiovascular Research, St. Elizabeth’s Medical Center, Tufts University School of Medicine, Boston, USA*

**Keywords:** EC-specific peptides, AAV, transduction

## Abstract

Vascular endothelial cells (EC) have been targeted for the treatment of pathological conditions such as atherosclerosis, hypercholesterolemia, post-angioplasty restenosis and hypertension. Non-pathogenic adeno-associated virus (AAV) has been shown as a good gene delivery tool in a variety of cell lines as well as in animal models. However, AAV has been reported to induce less endothelial cell transduction. AAV vector alone transduced HUVEC much lower than other cell lines including Hela, PAC1, and C2C12. Preincubation of AAV vector with EC membrane specific peptides markedly increased AAV transduction of HUVEC. On the contrary, those peptides did not affect AAV expression in other cell types. These EC-specific peptides may be a strategy for enhancement of AAV mediated-gene expression.

## INTRODUCTION

Vascular endothelial cells (ECs) are an attractive target for many-therapeutic applications, including targeting endothelium in atherosclerosis, hypercholesterolemia, postangioplasty stenosis, and hypertension (1-3). As adeno-associated virus (AAV) is a promising gene transfer vector for cardiovascular therapies (4, 5), AAV-mediated gene transfer to ECs is a highly inefficient and nonselective process (6, 7). Baker’s group (2001, 2004) recently demonstrated EC-specific peptides [SIGYPLP] (6), [MSLTTPPAVARP] (7) and [MTPFPTSNEANL] (7), which were isolated by linear phage display library method using human umbilical vein EC (HUVEC). Gratton *et al* (2003) reported that polybasic peptides derived from Drosophila antennapedia homeodomain or human immunodeficiency virus type 1 (HIV-1) transactivator protein (TAT) improved adenoviral and retroviral transduction in cultured monkey COS-7 cells, bovine aortic ECs and HUVECs (8). Accordingly, some peptides have a possibility to improve cellular uptake and therapeutic gene delivery of replication-deficient viruses in cells and *in vivo*. We thus conducted a series of experiments to verify the utility of the EC-specific peptide-complexed AAV vectors into ECs.

## MATERIALS AND METHODS

### AAV production and transduction assay

Standard serotype 2 AAV-CMV-lacZ vectors were prepared as described (9). The AAV vectors had a particle titer of 5 × 10^11^ to 2 × 10^12^/ml. Hela, PAC1 (smooth muscle cell), C2C12 (skeletal myoblasts) and HUVEC were cultured in DMEM supplemented and 1 × 10^3^ particles of AAV-CMV-LacZ per cell was infected to the cells. Forty eight hours after infection, the cells were fixed by 4% paraformaldehyde (PFA), and subjected to staining for beta gal activity (10). EC-specific peptides P1: [SIGYPLP] (6), P2: [MSLTTPPAVARP] (7) and P3: [MTPFPTSNEANL] (7) were synthesized by Tufts University Core Facility. AAV and each peptide were incubated overnight at 4 degrees and the complex was infected into cells following the same β-gal staining. Finally we counted the positive blue cells in each group (10).

### AAV genome assay

After cultured endothelial cells were infected with AAV-P1 complex or AAV alone, total DNA samples were prepared from the infected cells with Puregene DNA Isolation Kit (10) and using lacZ specific probes the samples were subjected for real- time (RT)-PCR for quantification of DNA expression as described before (7, 11).

### Statistical analysis

The mean and standard error (S.E.) were determined for multiple samples. ANOVA was performed to calculate the statistical significance. A *p* value of less than 0.05 was considered significant.

## RESULTS

**Figure 1:**
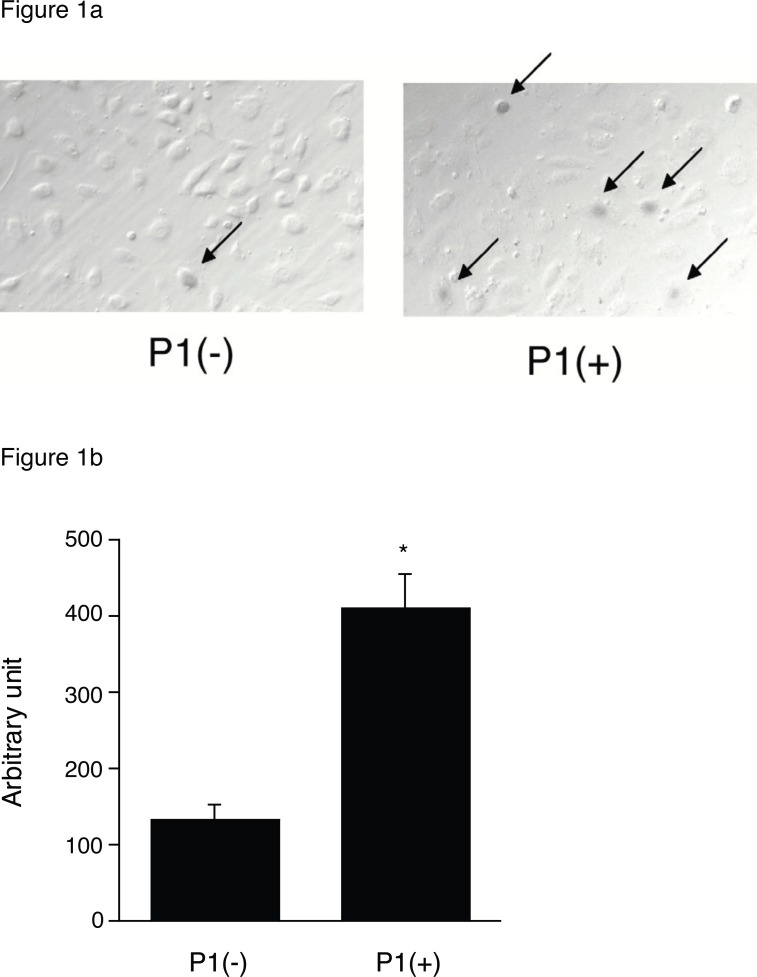
**a,** β-gal expression in AAV-infected HUVEC. 1 × 10^3^ particles titer of AAV-CMV-LacZ were incubated with or without 2 mg/ml P1, infected into HUVEC and 48 h after the cells were fixed and subjected to β-gal staining; **b,** RT-PCR assay for AAV genome. The AAV-infected HUVEC with or without P1 was harvested, and total DNA was subjected to RT-PCR assay using the lacZ specific primers. (n=4) *p<0.05 vs. P (-) control.

**Figure 2:**
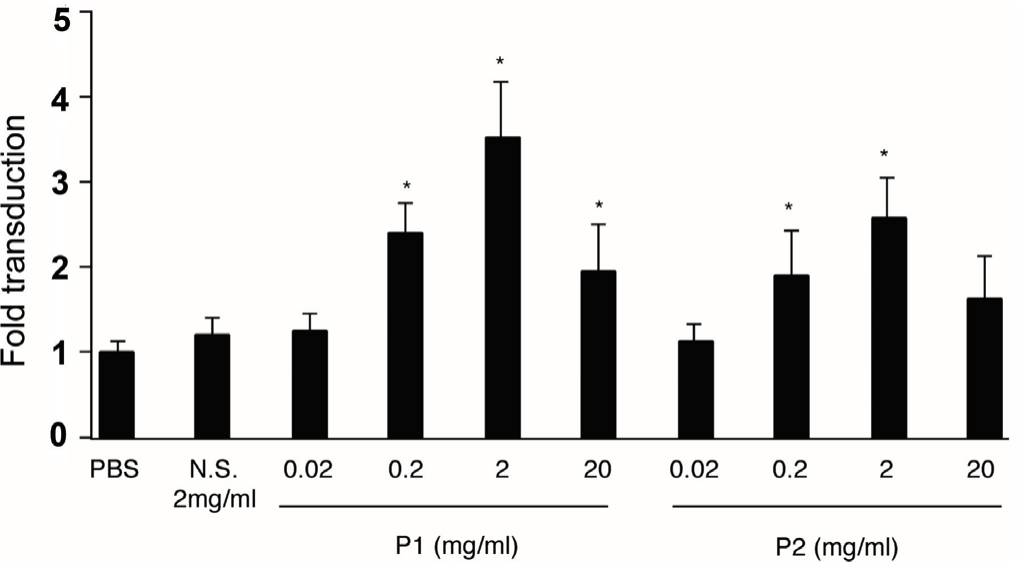
β-gal expression in AAV-infected HUVEC in a concentration dependent manner. N.S., P1 or P2 was incubated with AAV in the indicated concentration to make the complex before infection. 48 h after infection, the cells were fixed and subjected to β-gal staining. The β-gal-positive EC was counted. (n=4) *p<0.05 vs. PBS treatment control.

**Figure 3:**
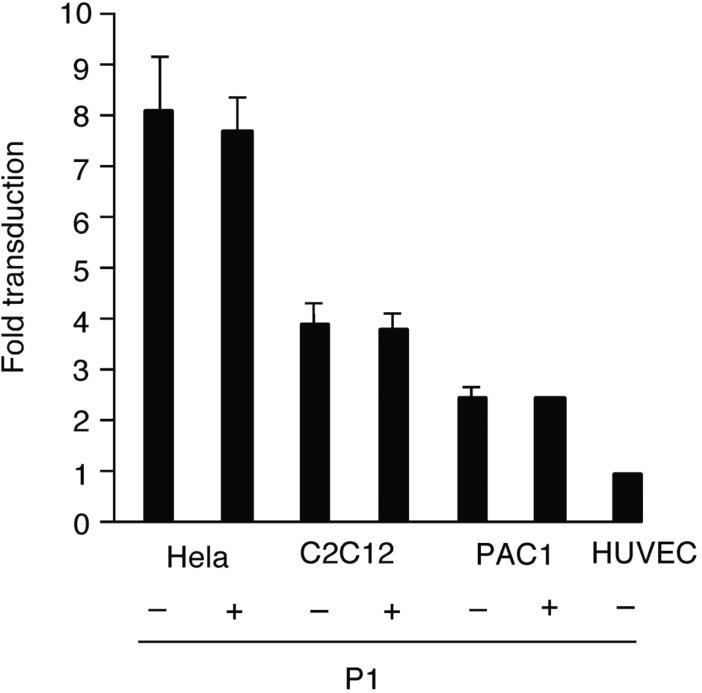
β-gal expression in Hela, C2C12, PAC1 with or without 2 mg/ml P1 peptide.

First, we examined whether certain EC-selective peptides complexed with viral particles, enhance AAV transduction of HUVEC. When AAV-CMV-lacZ was pre-incubated with P1, β-gal expression was increased compared to that of AAV alone as shown in Figure 1a. In addition, we examined the number of AAV genome in the infected HUVEC by quantitative RT-PCR. The AAV genome copies were markedly increased in the P1 (+) group compared to the P1 (-) group (Figure 1b). Next we determined which of the two peptides, P1 or P2, was more effective in inducing AAV transduction in ECs. After infection of AAV-CMV-lacZ complexed with P1 or P2 peptide, the infected HUVEC was subjected to β-gal assay in 48 h. While a non-endothelial specific (N.S.) peptide did not increase AAV transduction, both P1 and P2 peptides significantly transduced ECs in a dose-dependent manner at the range from 0.02 to 2 mg/ml (Figure 2). Quantitatively, P1 is more potent than P2 on AAV transduction in ECs (Figure 2). Next, we examined the specificity of these peptides by using other non-endothelial cell lines such as Hela, PAC1 and C2C12. Although AAV by itself induced β-gal expression in all cell types in the order of Hela, C2C12, PAC1 and EC (Figure 3), P1 did not augment β-gal expression in non-EC cell types (Figure 3). Moreover, when another 9-mer peptide, P3 [MTPFPTSNEANL], was compared with P2, β-gal expression by AAV-P2 complex remains higher than that of P3 (Table 1).

## DISCUSSION

In the present study, we tested a hypothesis that AAV transduction efficiency to vascular endothelial cells could be enhanced by complex formation of EC-selective peptide and viral particles. We found that peptides initially identified by Baker and colleagues using phage display to specifically targeting ECs are capable of increasing the AAV transduction efficiency specifically for ECs. This effect is thought to be at least in part due to an enhanced entrance of viral particle into the cell.

Recently, Gratton *et al* 2003 suggested that highly positively charged peptides can enhance the transduction by concentrating viral particles to the cell surface and by improving receptor-dependent uptake mechanism (8). The EC-specific peptides significantly enhanced AAV expression in ECs (Figure 1a, 2), and the number of AAV genome was also augmented by the peptides (Figure 1b). Since the EC-specific peptides hold an affinity to ECs, these peptides were thought to promote AAV vectors bind to the surface of endothelial cells and enter the cells, and to increase both AAV genome and transduction efficiency. In addition, the specificity of the peptide was preserved in only HUVEC (Figure 3), and as we tried to use another serotype 1 AAV vector, incubation with P1 also significantly augmented the transduction in ECs (data not shown). When we calculated the electric charge of peptides, P1 showed (±) even charge and P2 was a positive charge (+1) (Table 1). Because the result in Figure 2 indicates that P1 has a stronger effect on augmentation of AAV expression in HUVEC, electric charge may not be involved in uptake of AAV particles into ECs. In addition, since P1 is a 7-mer peptide and P2, P3 are 9-mer peptides, it suggests that the size is not related to the induction of AAV transduction. Both peptides revealed induction of AAV in a concentration-dependent manner and the concentration more than 2mg/ml conversely decreased AAV expression (Figure 2). It is possible that excess peptide may decrease the number of AAV particles attaching with it.

**Table 1:** Correlation of peptide charge and AAV transduction ratio

Peptide sequence	Electronic charge	AAV transduction

P1: SIGYPLP	0	1
P2: MSLTTPPAVARP	+1	0.69
P3: MSLTTPPAVARP	-1	0.48

The mean of AAV transduction ratio is shown when we regard P1 as a control.

We also performed *in vivo* assay using mouse or rat blood vessels. When we injected the complex of AAV and the P1 peptide from the tail vein, no β-gal positive ECs were observed in the aortic artery (data not shown). It is conceivable that the ECs of artery could hardly uptake the AAV-protein complex compared to cultured ECs. Alternatively, the AAV-P1 complex formed *in vitro* may be not stable when delivered *in vivo*. Baker’s group showed the peptide-modified AAV, which includes the specific peptide was genetically incorporated into AAV capsid protein sequence (6, 7). That vector should be used for targeting EC expression of *in vivo* use. Further studies are required to evaluate the binding strength between electric charge of protein and AAV.
